# A geopolitical turning point? Enlargement discourse after the Russian invasion of Ukraine

**DOI:** 10.1177/14651165251367355

**Published:** 2025-08-21

**Authors:** Tom Hunter, Natasha Wunsch, Marie-Eve Bélanger

**Affiliations:** 1 Department of Political Science, 27217 University of Zurich, Zürich, Switzerland; 2 Department of European Studies, 27211 University of Freiburg, Fribourg, Switzerland; 3 Department of Political Science and International Relations, University of Geneva, Geneve, Switzerland

**Keywords:** European Parliament, enlargement, Ukraine, rhetorical entrapment, text analysis

## Abstract

Russia's full-scale invasion of Ukraine in February 2022 has been cast as a geopolitical turning point for European Union enlargement. Whilst solidarity with Ukraine has translated into the swift granting of candidate status only a few months later, we know less about how the European Union's position towards other candidates has evolved. Drawing on the concept of ‘rhetorical action’, we explore the evolution of enlargement discourse in the wake of Russia's aggression: does the European Union's renewed commitment to enlargement concern Ukraine alone, or rather expand beyond Kyiv to signal a broader revival of the accession process? And how do Members of the European Parliament’s evolving positions on the pursuit of European Union widening align with prevalent ideological and geographic cleavages in the European Parliament? Analysing an original dataset of over 1700 hand-coded enlargement statements from the European Parliament's ninth term (2019–2024), we show that Russia's full-scale invasion coincides with a generalised reinvigoration of the European Parliament's enlargement discourse that is driven in particular by a shift in mainstream parties’ positions and by Members of the European Parliament from Central and Eastern Europe. However, the effects of this ‘rhetorical entrapment’ remain limited to Members of the European Parliament from mainstream party groups, with radical right actors instead avoiding to engage in enlargement-related debates.

## Introduction

Russia's full-scale invasion of Ukraine in February 2022 sent shockwaves through the European Union (EU). Besides supplying weapons and aid, the EU also revised its political stance towards Kyiv. At a European Council meeting just 4 months after the beginning of Russia's aggression, Ukraine was granted EU candidate status amid a climate of solidarity and goodwill in which the Commission President described the country's future as being ‘in our Union’ (Von der Leyen, 2023). Yet this swift formal progress on the path to accession was not universally applauded: whilst some media outlets argued that the invasion had given enlargement a whole fresh impetus and a ‘new lease of life’ (Economist, 2023), others reported extensively on the ‘Balkans’ frustration over Ukraine's fast-track to EU membership’ (Financial Times, 2023).

Against this backdrop, we examine the recent developments in the enlargement process in light of the concept of ‘rhetorical action’ (Schimmelfennig, 2001, 2021) first posited in the context of the EU's 2004 Eastern enlargement. Rhetorical action implies ‘the strategic use of norm-based arguments’ to convince others of the legitimacy of a certain course of action even against their initial reluctance (Schimmelfennig, 2001: 48). ‘Rhetorical entrapment’ ensues where the underlying norms mobilised to justify a proposal are shared within a community, thus forcing opponents first to accept the proponents’ claims and then to act accordingly (Leuffen et al., 2024: 8 and 27). Drawing on this notion of rhetorical action and its resulting rhetorical entrapment, we investigate empirically whether the sudden shift in political support for Ukraine's membership perspective in the wake of Russia's full-scale aggression has provided an opportunity for pro-enlargement actors to push for a broader revival of EU enlargement policy after February 2022. Is renewed support for enlargement after the Russian invasion limited to Ukraine alone, or do we observe a generalised reinvigoration of enlargement policy?

We focus our analysis on the European Parliament (EP) as one of the central institutions involved in the EU accession process and analyse how Members of the European Parliament (MEPs) have positioned themselves in enlargement-related debates during the mandate that encompasses Russia's full-scale invasion of Ukraine. The heightened geopolitical tensions in Europe following the Russian aggression have produced a wave of new scholarship that has examined the ways in which Russia's attack on Ukraine has shaped public opinion (Panchuk, 2024), the nature of the enlargement process (Anghel and Džankić, 2023), and the deepening of European integration more generally (Genschel et al., 2023). Our analysis adopts a new angle by focusing on enlargement *discourse* in the wake of Russia's all-out attack. To examine the evolving rhetorical patterns in enlargement discussions, we analyse an original corpus of over 1700 hand-coded statements collected from plenary debates on enlargement throughout the EP's ninth session (2019–2024). The EP is a particularly relevant arena to study enlargement discourse given the need for its formal approval of any new accession. At the same time, the ideological and geographic diversity of its membership enables an analysis of the political dynamics that underpin discursive changes in the wake of Russia's aggression.

Three main theoretical expectations guide our analysis: first, we expect enlargement discourse as a whole to become more supportive after February 2022. We hypothesise that following the shift in position towards Ukraine's membership bid, the need for rhetorical consistency makes it difficult for MEPs to support Ukraine's accession perspective without extending this support to similar candidate countries, thus contributing to a renewed discursive commitment to the enlargement process as a whole. Second, we assume MEPs’ ideological orientation to affect their positioning. Concretely, we expect MEPs from mainstream political groups to be more likely to respond to norm-based arguments in favour of enlargement, whereas we hypothesise Eurosceptic challengers to employ a strategy of ‘avoidance’ (De Vries and Hobolt, 2020) as their anti-enlargement position becomes more incongruent with public opinion. Finally, we anticipate the increased salience of enlargement to be driven in particular by MEPs from Central and Eastern Europe (CEE), who have traditionally been more supportive of enlargement in light of their own more recent accession experience as well as the geographic proximity of the candidate countries.

Our empirical findings lend support to our expectation of a generalised increase in both salience and support for enlargement in the wake of Russia's full-scale aggression against Ukraine. Although Ukraine becomes the single most discussed individual candidate after the invasion, it does not displace the Western Balkans, which remain the most discussed region overall. Besides, Moldova and Georgia, as the two Eastern Partnership candidates that most clearly share Ukraine's EU ambitions, benefit most from increased support for enlargement after the invasion. We also confirm that MEPs’ ideological orientations shape their positions on enlargement: a marked increase in support for enlargement among mainstream party groups after the invasion contrasts with the discursive behaviour of radical left and radical right parties, who remain broadly sceptical of enlargement and tend to disengage from enlargement debates. Finally, our analysis lends support to our expectation that the increased salience of enlargement is driven in particular by MEPs from CEE, underlining the geographic dimensions of the EU's newfound solidarity with its Eastern neighbourhood.

Our analysis makes several contributions to the literature on enlargement in general and the impact of Russia's full-scale invasion in particular. First, we show that the fundamental changes in the geopolitical context trigger a dynamic of ‘rhetorical entrapment’, whereby Russia's attack allows enlargement supporters to frame Ukraine's accession in both moral and strategic terms and to argue in favour of extending this support to additional candidate countries. This reverses earlier trends of a gradual hardening of EP discourse on enlargement also among pro-European parties in light of radical populist contestation of further widening (Bélanger and Wunsch, 2022; Wunsch and Bélanger, 2023). Rhetorical action in favour of enlargement appears particularly effective in the case of the Eastern Partnership countries that share Ukraine's pro-European orientation, suggesting that geographic proximity and domestic support for the accession perspective as important scope conditions for the successful use of norm-based arguments in the enlargement context. Second, we show how mainstream MEPs increase their support for Ukraine's prospects after the invasion in a way that largely matches public opinion (Hoffmann, 2023). We contribute to research analysing how radical populist parties have reacted to the invasion (Braghiroli, 2023; Ivaldi and Zankina, 2023) by identifying a strategy of ‘avoidance’ (De Vries and Hobolt, 2020) after their openly enlargement-hostile positions have become incongruent with public opinion. Finally, our findings hold important practical implications with regards to the future of EU enlargement. Specifically, since radical right parties made significant gains at the 2024 EP elections and are already at the helm of several states in the Council, the fact that these parties’ low levels of support remain unchanged post-invasion may be a source of worry for proponents of enlargement. In this sense, it remains an open question whether the renewed discursive support that has once again placed enlargement as a crucial item on the EU's agenda will eventually translate into concrete steps towards further accessions.

## Enlargement discourse after the Russian invasion

A growing literature has begun to speculate what Russia's full-scale invasion of Ukraine might mean for the EU enlargement process and European integration more generally. Scholars have expressed scepticism as to whether candidates whose progress on the path towards accession was unusually fast in the early stages will ever access full membership (Anghel and Džankić, 2023), or whether the war will lead to a more federal EU (Genschel et al., 2023). Others have argued that the invasion is serving as an engine of integration, particularly with respect to the emergence of the Commission as a geopolitical actor (Håkansson, 2024). What is clear is that the speed of the EU's decision-making in the aftermath of the invasion has been unprecedented. Ukraine in particular saw its accession process move from candidate status to the opening of negotiations in less than 2 years. Public opinion studies also show how support for Ukraine's candidacy increased after the invasion (European Council on Foreign Relations, 2023; Panchuk, 2024; YouGov, 2022). Panchuk in particular concludes that ‘a certain permissive consensus concerning Ukraine's entry in the EU may have developed’, but that for other candidates ‘constraining dissensus in public preferences is still the order of the day’ (2024: 38).

In this article, we build on this existing work by focusing on enlargement *discourse* in the EP and how this has evolved since Russia's invasion. There are several reasons why this discourse matters. First, the EP is a direct decision-maker in the enlargement process and its approval is required before a country can join. Second, political parties are influential cue-givers in debates about European integration (Hooghe and Marks, 2005), and could therefore shape how the public feels about EU enlargement in a way that grants governments more (or less) leeway when negotiating enlargement in the Council. Importantly, whilst existing studies have explored enlargement discourse in both national and European settings (see Bélanger and Wunsch, 2022; Economides et al., 2024; Wunsch and Bélanger, 2023; Wunsch and Olszewska, 2022), their investigation periods precede February 2022 and therefore cannot address the effects of the Russian invasion.

### Rhetorical entrapment and the need for consistency

The concept of ‘rhetorical entrapment’ (Schimmelfennig, 2001, 2021) has become central to understanding the process of EU enlargement. According to this account, enlargement dynamics cannot be captured through a rationalist lens based on cost–benefit analyses alone. Instead, members of the international community can be shamed into supporting positions that go against their preferences through the strategic use of norm-based arguments. Because members of the community care about their reputation, they worry about the credibility of their arguments, which depends on both impartiality and consistency (Elster, 1992).

Whilst rhetorical entrapment is often associated with the need for consistency between words and actions (Scherzinger, 2023), another central pillar to Schimmelfennig's seminal article is the need for consistency in the arguments themselves used to make the case for (or against) enlargement. For instance, Schimmelfennig notes that ‘actors must avoid creating the impression that they use value and norms cynically and *inconsistently*’ and that ‘the requirement of consistency applies *both* to the match between arguments and actions and to the match between arguments used’ (2001: 65, emphasis our own). In other words, it is very difficult for a politician who cares about their reputation in the European community to support the prospects of one candidate that ticks certain boxes of the accession criteria whilst opposing the prospects of another candidate that ticks the very same boxes.

Applying the concept of ‘rhetorical action’ in the wake of Russia's full-scale invasion of Ukraine, we argue that it is precisely this need for rhetorical consistency that makes it difficult for politicians in the EU to be supportive of Ukraine's enlargement prospects without also extending this support to other candidates. Not only would this allow current candidates to point out the hypocrisy in this position; it would also enable existing members of the European community, such as MEPs from other party families (or even from within their own party group^1^) to highlight inconsistencies in their rhetoric. In particular, such behaviour would expose MEPs to accusations of double standards and favouritism towards Ukraine that may undermine the credibility of the enlargement process as a whole. In a recent special section that discusses the enduring relevance of rhetorical action in the changed, post-2022 geopolitical context, Schimmelfennig himself argues that the conditions for rhetorical entrapment have become more favourable since Russia's full-scale aggression (Leuffen et al., 2024: 37). Whilst the technocratic nature of the enlargement process and the lack of compliance in the Western Balkan countries made it harder to mobilise normative arguments in favour of their accession, the shift in focus ‘from transformation deficits to political commonalities and from internal disagreements to external unity’ following Russia's all-out aggression offers more fertile ground for political actors seeking to argue in favour of a pursuit of enlargement with reference to candidate countries’ adherence to shared values (Leuffen et al., 2024).

Yet, it remains an open question to what extent this newfound solidarity extends beyond Ukraine to other countries in Europe's East. Specifically, we do not consider all candidates to be likely to benefit equally from the need for consistency and thus the potential for rhetorical entrapment. Instead, political actors’ ability to highlight inconsistencies between the treatment of Ukraine and another candidate should be particularly potent when that country shares many characteristics with Ukraine. In particular, we expect other countries from the EU's Eastern Partnership to benefit most from renewed support for enlargement in the wake of Russia's invasion of Ukraine. As states that are also located in Russia's immediate neighbourhood – and therefore under its threat – these countries are the most likely to be associated with the Ukrainian cause in the minds of the public and policy-makers. More generally, the desire of the EU to position itself as a geopolitical power vis-à-vis Russia (Anghel and Jones, 2023) that has driven Ukraine's accelerated accession talks is particularly relevant for these countries.

At the same time, we also make a distinction within the Eastern Partnership region between those countries that explicitly share Ukraine's EU ambitions, and those more authoritarian-leaning ones that do not. Indeed, whilst Georgia and Moldova's ‘European perspective’ had long been recognised prior to the invasion, this is not the case for Belarus and Azerbaijan. Belarus’ leader, Aleksandr Lukashenko, has been labelled ‘Europe's last dictator’ (Brel-Fournier and Morrison, 2021) and has long embraced closer relations with Russia than with the EU (Newnham, 2020), with Belarus eventually suspending its participation in the Eastern Partnership in June 2021. Azerbaijan also famously rejected an Association Agreement with the EU in February 2016. The case of Armenia is more complex given its recent declaration of interest in seeking EU membership following Azerbaijan's violent retaking of the contested region of Nagorno-Karabakh. Still, this new development falls into the very end of the time period we investigate and we therefore do not expect to see Armenia benefit from increased support for enlargement following Russia's invasion in the same way as those Eastern Partnership countries that had made their European perspective a long-standing priority.

In sum, we consider the changed geopolitical context following Russia's full-scale invasion to offer potential for a renewed relevance of rhetorical action as a means to build political support for further enlargement. Due to the potential accusation of hypocrisy and inconsistency that represent central elements of the mechanism of rhetorical entrapment, we expect the strategic mobilisation of normative arguments by pro-enlargement actors after February 2022 to translate into a broader support for enlargement not just for Ukraine, but beyond. We anticipate this increased support to be particularly strong for candidates most similar to Ukraine, namely those Eastern Partnership countries that share Kyiv's European aspirations. Our first set of hypotheses is thus:

*H1a:* MEPs’ increased support for enlargement after the invasion extends beyond Ukraine.

*H1b:* The increase in supportive discourse is more pronounced for Eastern Partnership candidates that share Ukraine's EU ambitions than for other candidates.

### Ideological orientation: Mainstream versus radical parties

Ideology is a key differentiator in MEPs’ positioning on EU policies in general and enlargement in particular (Hix et al., 2005; Wunsch and Bélanger, 2023). Accordingly, we expect the effect of the invasion on enlargement discourse to vary depending on MEPs’ ideological orientation. Concretely, one of the scope conditions for rhetorical action is the adherence to common norms from which political actors derive both domestic and international legitimation (Leuffen et al., 2024: 12). Accordingly, we expect MEPs from mainstream European party groups (EPGs)^2^ to be more susceptible to rhetorical entrapment than their more radical counterparts.^3^ Contrary to radical EPGs that question the EU's legitimacy and are therefore less bound by the norms of the European community, the EP's mainstream groups are considered drivers of the EU's ‘normative voice’ and its emphasis on values (see Feliu and Serra, 2015). As actors that are (or at the very least, have claimed to be) sensitive to the values and principles of the EU, they are therefore particularly bound by these principles and are likely to find it difficult to support Ukraine's enlargement prospects without also supporting the prospects of other candidates that share its characteristics.

For mainstream EPGs, the change in public mood following Russia's full-scale invasion of Ukraine strengthens the hand of pro-enlargement MEPs to overcome the long-standing enlargement ‘fatigue’ (see Economides, 2020; O’Brennan, 2014; Szolucha, 2010) that had made the further widening of the EU a divisive issue. Whilst earlier findings pointed to a declining discursive support for enlargement among mainstream MEPs despite continued support in roll-call votes (Wunsch and Bélanger, 2023), the growth in public solidarity towards Ukraine is likely to re-energise and embolden those factions of these parties that have always been in favour of a widening of the EU, resulting in a clearer rhetorical commitment to enlargement among mainstream party groups.

In contrast, we suggest that the increased goodwill towards Ukraine seen in public opinion surveys throughout the continent (Panchuk, 2024; YouGov, 2022) places radical party groups in a delicate position when discussing enlargement. These parties build their electoral success – particularly in European contests – upon mobilising Eurosceptic positions that are sceptical of both deepening and widening European integration (see Hobolt and Tilley, 2018; Kriesi, 2016). Yet increased support for Ukraine and its EU candidacy suddenly places these parties’ positions at odds with the public mood. This case of an unexpected event changing public opinion on an EU issue, and therefore suddenly presenting Eurosceptic parties with a dilemma, can be compared to Brexit, which placed radical parties in a difficult position once the negative impacts of a policy they had long advocated for became clear (Hunter, 2025; Martini and Walter, 2024).

We therefore expect radical EPGs to respond to the increased support towards Ukraine after the invasion with a strategy of ‘avoidance’ (see De Vries and Hobolt, 2020; Green-Pedersen and Mortensen, 2015). Rather than fundamentally changing their sceptical positions towards EU enlargement, we instead anticipate them to downplay the issue by mentioning it less in their parliamentary speeches or choosing not to participate in debates at all. This allows them to avoid highlighting incongruity with the public mood. Radical right parties in particular have long advocated a stop to EU enlargement, linking it to their concerns around immigration and free movement (Heinisch et al., 2020). We therefore expect the effect to be strongest for radical right parties. Our hypotheses on the differing effects of ideology upon MEPs’ enlargement discourse are therefore:

*H2a:* For mainstream party families in the European Parliament, enlargement discourse becomes more salient and more supportive after the invasion.

*H2b:* For radical party families in the European Parliament, enlargement discourse becomes less salient and equally (un)supportive after the invasion.

*H2c:* The strategy of avoidance is most pronounced for radical right party groups compared to radical left party groups.

### Geographic origin: East versus West?

Finally, we consider to what extent an MEP's country of origin is likely to shape her position towards enlargement post-invasion. Whilst scholars have concluded that the EP is in many ways a ‘normal’ parliament (Hix et al., 2005), with MEPs more likely to vote in line with fellow party members than with fellow nationals, for certain issues, national origin is also a strong predictor of MEPs’ positions (Hix et al., 2005). Enlargement has been shown to be one such issue (Bélanger and Schimmelfennig, 2021; Wunsch and Olszewska, 2022). Scholars have shown how attitudes towards enlargement differ significantly among the newer member states that joined with the ‘big bang’ enlargement of 2004/2007 and older member states from Western and Northern Europe. For instance, research from Toshkov et al. (2014) shows that whilst attitudes towards enlargement across the continent have become less supportive since 2004, net support among the newer member states from CEE remains significantly higher.

This trend is confirmed by more recent polling from the European Council on Foreign Relations (ECFR). A survey conducted in November 2023 in six member states found significantly higher support among newer CEE member states than among older member states. For instance, whilst 51% of Romanian respondents and 48% of Polish respondents think the EU ‘should be looking to add new member states at this moment in time’, the share of respondents expressing the same view in Denmark, France, Germany, and Austria is between 27% and 29% (ECFR, 2023). As both instrumental and identity reasons are important to explain attitudes towards further enlargement (Azrout et al., 2013; Dixon, 2010; McLaren, 2007), it is perhaps unsurprising that the newer members, who share stronger economic and cultural ties with current candidates, are more supportive of their eventual accession.^4^

It is worth noting that Stolle (2024) finds that CEE countries are actually less supportive towards Ukraine specifically, at least for certain measures that impose direct costs such as sending troops or accepting higher energy costs because of sanctions. Our argument, however, focuses rather on how the invasion impacts general positions towards enlargement and is also exclusively interested in the accession of candidates, whereas Stolle considers a range of measures, including the admission of refugees, sending humanitarian aid, and providing additional weapons and military equipment.

Overall, we expect the differences in general attitudes towards enlargement to also be reflected in enlargement discourse. As MEPs from CEE are typically more supportive of enlargement in general, the situation in Ukraine gives them the opportunity to once again place enlargement at the top of the agenda. We anticipate this reality to translate not simply into increased levels of support in the discourse, but also into efforts to raise the salience of enlargement in the EP by mentioning it more frequently in their speeches. Our final set of hypotheses is therefore:

*H3a:* The increased salience of enlargement discourse post-invasion is more pronounced for MEPs from Central and Eastern Europe.

*H3b:* The increased support in enlargement discourse post-invasion is more pronounced for MEPs from Central and Eastern Europe*.*

## Research design

### Statements about enlargement in EP debates on EU membership

To test our hypotheses, we draw on an original dataset of statements made by MEPs during debates on enlargement throughout the ninth Session of the European Parliament (2019–2024). A statement is defined as the MEP's position on a country's membership to the EU, supported by arguments. Positions are systematically coded to indicate whether MEPs express support, conditional support, or opposition to a country's EU membership. Each statement also captures the candidate country (or region) being discussed.

In the first step, our data collection involved identifying all debates in the EP's ninth session that mentioned either the name of a candidate country (or region) or referred to enlargement in their title. From these debates, we extracted individual statements whenever an MEP took a clear stance on the enlargement prospects of a candidate country or region. Notably, a single speech can include multiple statements if the MEP references more than one candidate. For example, if an MEP expresses support for Ukraine's membership, continued enlargement prospects for the Balkans, and opposition to Turkey's accession in one speech, we would code this intervention as three distinct statements.

To validate our coding, we assessed the intercoder reliability using Cohen's kappa for a subset of speeches coded by a research assistant as well as one of the authors and found high levels of agreement. Specifically, when analysing the alignment between coders across three indicators – identification of a statement, position, and arguments – we achieved a kappa value of 0.80. This score is considered substantial, indicating a strong reliability of our coding process.

Next, we conducted a comparative analysis of statements made before and after the Russian invasion of Ukraine on 24 February 2022. From a research design perspective, the date is helpful, as it falls almost exactly halfway through the EP's ninth session. We identified 568 enlargement statements before the invasion and 1139 after, providing us with a large sample to test whether and how enlargement discourse in the EP changed after Russia's attack. Table 1 displays examples of statements for each of the three positions, illustrating how speeches sometimes reference individual candidates and at other times refer to a group of candidates or a region. The full codebook can be found online as part of the article's replication materials (Supplemental materials).

**Table 1.** Examples of coded enlargement statements in the European Parliament.

Speaker	Date	Statement	Position
Viola V. Cramon Taubadel (Greens, DE)	2019.10.23	‘To continue to keep the door to the EU closed for Albania and North Macedonia is not only an affront to the governments of these countries but above all to the young people of these countries. With this decision not to open accession despite the criteria already being fulfilled, these opportunities for economic transformation and domestic reforms remain on hold. Others will not wait long to fill the gaps in the Western Balkans left by the EU. In particular, Russia, Turkey, China, Qatar, and Saudi Arabia are actively seeking political cooperation in the region. This can hardly be in our interest’.	Support
Andreas Schieder (S&D, AT)	2023.07.11	‘Over the past decade, Albania has implemented a tremendous number of reforms – bureaucratic reforms, rule of law reforms, judicial reforms, the fight against corruption, and even at the local level, such as in Tirana, where concrete measures have significantly improved quality of life and urban development. Having said that, one might ask: Is everything perfect? No. Albania still needs to undertake many more reforms. Environmental issues, climate concerns, and sustainability are major topics, as well as labor rights and unions. But one thing is clear: Cooperation with Albania, especially in the context of enlargement, is effectively driving important reforms forward in a professional manner’.	Conditional support
Philippe Olivier (ID, FR)	2022.11.22	‘You cannot work together as 27 and yet you want to expand further… The extension you propose is an endless expansion, characteristic of an empire, an empire named the EU. However an empire implies imperialism, which means the idea of subjugating peoples to a higher order, a central authority, an empire which in this case is a technocratic nebula serving a market ideology’.	Oppose

### Empirical strategy

To test our hypotheses statistically, we estimate a range of ordered logistic regression models. Our unit of analysis is the statement, and our dependent variable is an (ordered) categorical variable with ‘oppose’ as the lowest value, ‘conditional support’ as the middle value, and ‘support’ as the highest value. Our key explanatory variable, ‘post-invasion’, is binary and takes a value of 1 if the speech takes place after 24 February 2022. Whilst the structure of our data and research design do not allow us to make direct causal claims about the impact of the invasion on discourse, we are able to test whether differences before and after this date are statistically significant.

Importantly, our data is hierarchical or ‘nested’ (Zuur et al., 2009), with positions nested within speakers, who are nested within party groups. We therefore run multilevel ordered logistic models with random effects for speakers and party groups.^5^ We test the appropriateness of random effects in a number of ways. First, we compare the Akaike information criterion (AIC) and Bayesian information criterion (BIC) scores of random effects models with simpler fixed-effect only models. We find that in each case, the random effects model has significantly lower AIC and BIC scores, which means that the inclusion of random effects is indeed appropriate. We then calculate the intraclass coefficient (ICC) for all the random effects models. We find that the ICC is consistently high, ranging from 0.471 to 0.943. These high ICC values highlight the strong clustering in the data and justify the inclusion of random effects. Table A1 in the Online appendixppendix displays the ICC, AIC, and BIC for all models. As we are using ordinal logistic regression, we also tested the proportional odds assumption using the Brant test, which indicated no significant violations (*p* = 0.45).

## Results

### Increased support after the invasion

**Figure 1.**
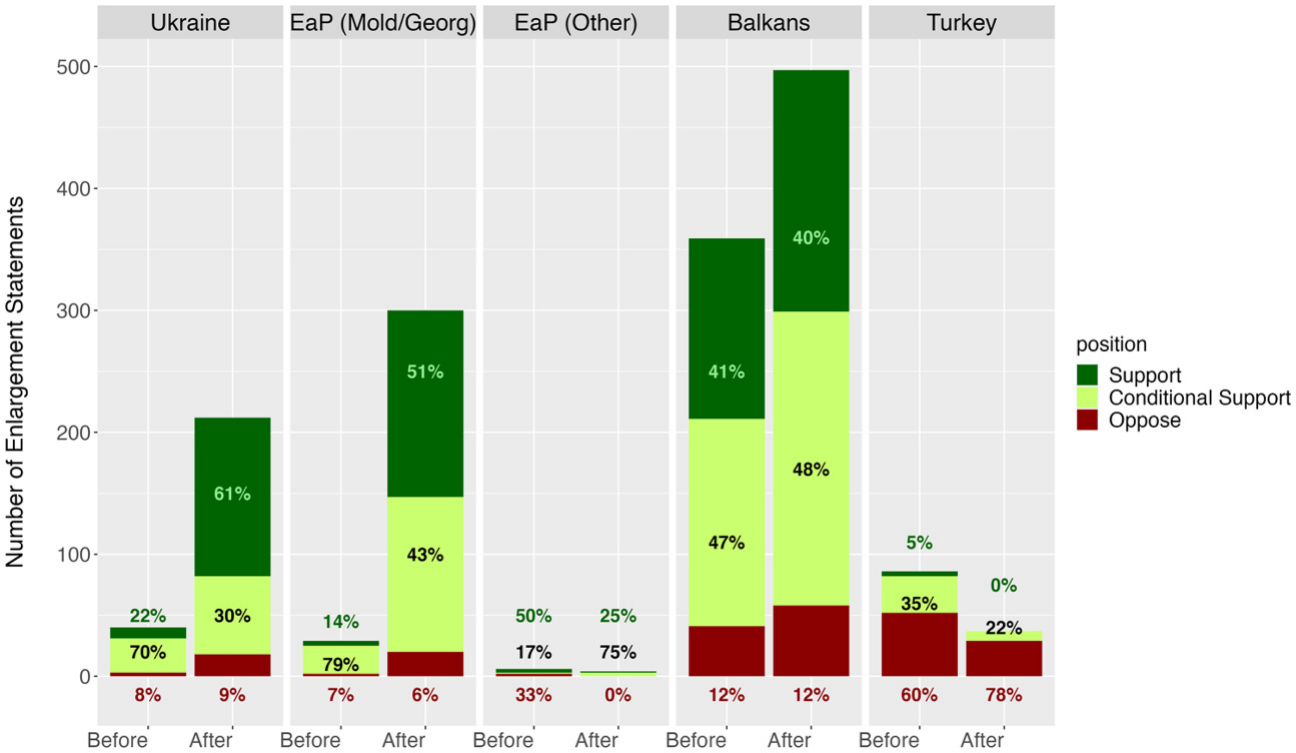
Position by candidate country/region before and after the Russian invasion.

*Note*: Percentage labels refer to share of enlargement statements in the respective investigation period (before and after the invasion) coded as ‘Oppose’, ‘Conditional Support’, and ‘Support’.

Our overarching theoretical assumption was that more support for enlargement would not simply be limited to Ukraine, but that other countries would also benefit from a more positive discourse around further accessions. Furthermore, we hypothesised that the increase in support would be more pronounced for Eastern Partnership candidates that share Ukraine's EU aspirations. In Figure 1, we plot the number of statements made about the enlargement bids of Ukraine, the Eastern Partnership countries^6^ (distinguishing between Moldova and Georgia that share Ukraine's EU aspirations, and the three others), the Western Balkans, and Turkey. The figure shows that whilst the number of statements about Ukraine increased the most, making Ukraine the single most discussed candidate country after the invasion, it does not dominate enlargement discourse to the point of crowding out other candidates.

Instead, the magnitude of the increase in statements about Ukraine after the invasion is approximately the same as the increase for Moldova and Georgia. Furthermore, the number of enlargement statements about Western Balkan candidates also increases significantly, with discourse about the Balkans region remaining clearly dominant as well as largely supportive both before and after the invasion. The big loser is Turkey, for which the number of statements more than halves after the invasion, with discourse becoming even more dominated by statements opposing its accession to the EU. We also notice how the prospect of rhetorical entrapment does not extend to those Eastern Partnership countries whose EU ambitions are less clear: Azerbaijan, Armenia, and Belarus are barely mentioned in enlargement discourse throughout the entire period under investigation.

To test our hypotheses about position changes statistically, Table 2 presents the results from multilevel ordered logistic regression models.^7^ The unit of analysis is the statement, and we run the model first without candidate country dummies (model 1), then with candidate dummies (model 2), and finally with an interaction between candidates and our post-invasion dummy (model 3). The reference category for these models is the Western Balkans. The models include random effects for party groups and speakers. Results show how discourse for enlargement becomes more supportive in general and that this effect is driven not only by Ukraine but also by increased support for the Eastern Partnership candidates that share Ukraine's EU ambitions. These findings are also borne out descriptively: Figure 1 shows how for Ukraine and Moldova/Georgia, the mode (most common category) moves from ‘conditional support’ to ‘full support’. For the Western Balkan candidates, there is no statistically significant difference in the nature of discourse before and after the invasion. In both cases, the discourse is broadly positive, with ‘conditional support’ as the mode.^8^

**Table 2.** Ordered logistic regression results with interaction effects (DV = Position).

	Model 1	Model 2	Model 3
Post-invasion	0.55***	0.46***	−0.06
	(0.14)	(0.16)	(0.18)
Ukraine			−0.67
			(0.42)
Eastern Partnership			−0.54
			(0.35)
Turkey			−3.73***
			(0.38)
Ukraine × Post-invasion			1.63***
			(0.47)
Eastern Partnership × Post-invasion			0.91**
			(0.39)
Turkey × Post-invasion			−1.06
			(0.65)
Observations	1600	1600	1600
Party RE	Yes	Yes	Yes
Speaker RE	Yes	Yes	Yes
Candidate country FE	No	Yes	No

*Note*: The reference category for models 1 to 3 is the Western Balkans.

*p < 0.1; **p < 0.05; ***p < 0.01.

**Figure 2.**
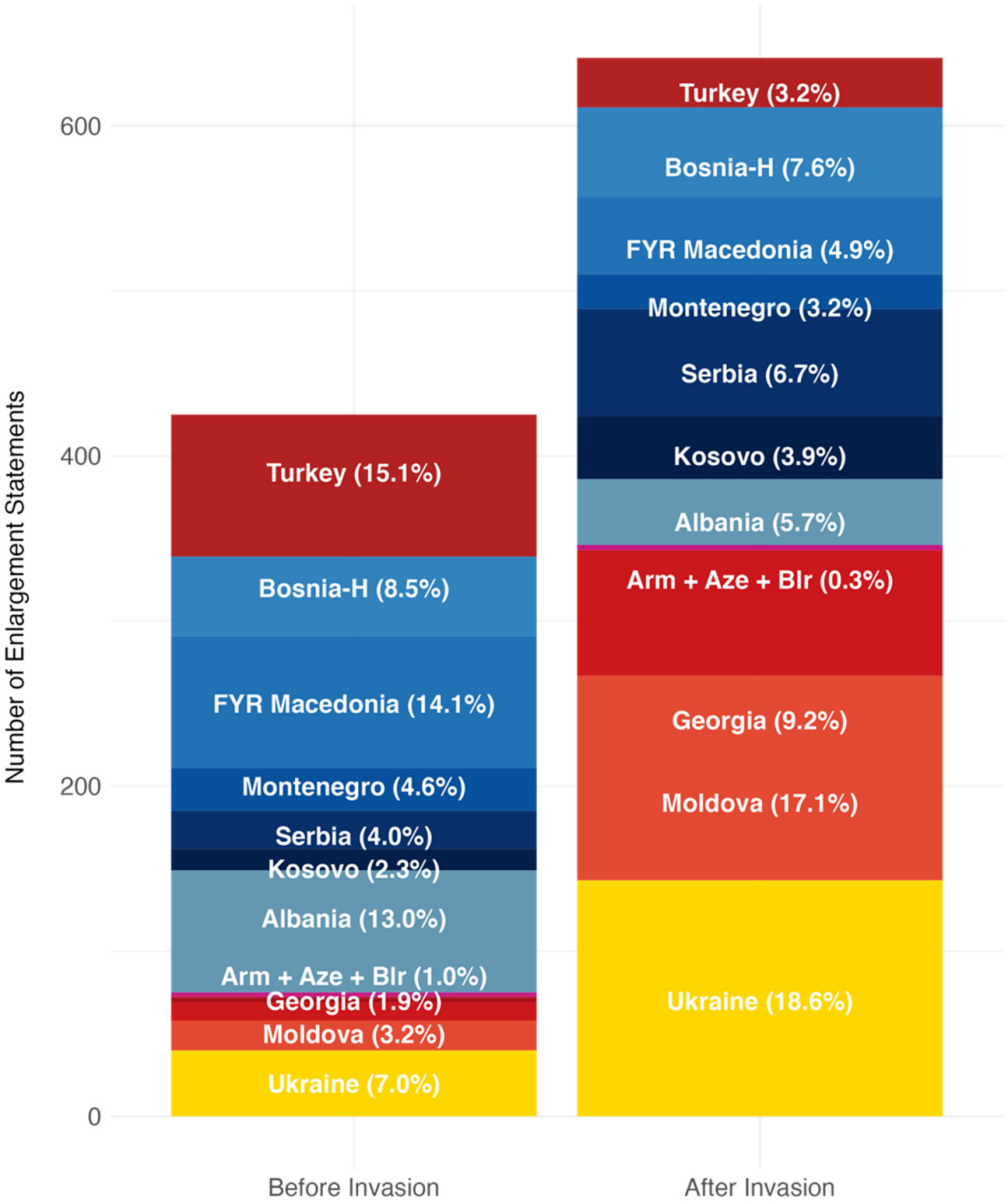
Which candidate countries are discussed the most in the EP ninth session?

*Note*: Percentages calculated by dividing the number of statements on each explicitly named candidate by the total number of enlargement statements in the respective investigation period (before and after the invasion). Note that our sample includes general statements about enlargement and statements about regions (e.g. ‘Western Balkans’) which are not included in this figure (and therefore the percentages in each stacked bar chart do not add up to 100%). EP: European Parliament.

To provide further evidence for the fact that the increase in support for enlargement extends beyond Ukraine, Figure 2 plots the number (and share) of statements recorded for each candidate country before and after the invasion. The table is ordered by the total number of statements across the EP's ninth session. Whilst Ukraine is overall the most mentioned candidate country, we show that even after the invasion, approximately 81% of all enlargement statements were made about countries other than Ukraine.

Overall, we thus conclude that there is little evidence of Ukraine enjoying a monopoly over the EP's increased discursive support for enlargement. Instead, our findings confirm both our hypotheses on general trends, with enlargement discourse post-invasion becoming more supportive in general (H1a) and this increase being most pronounced for Georgia and Moldova (H1b). Whilst the salience of Ukraine's candidacy unsurprisingly increases after the Russian invasion, so does that of Eastern Partnership countries that share Kyiv's EU aspirations. Besides, the Western Balkans remain the most discussed enlargement region, with fears that they would be sidelined following Russia's invasion thus appearing misplaced.

A closer qualitative look at the enlargement statements themselves provides evidence for the mechanism of rhetorical entrapment being at play. Indeed, there are many examples of rhetoric that present equivalence between Ukraine, Georgia, and Moldova and (attempt to) shame other actors who support Ukraine into also supporting the prospects of these additional candidates. For instance, in a statement at an EP debate on Eastern Partnership countries, Łukasz Kohut, a Polish MEP from the Social Democrats (S&D), declared that ‘For them [Georgia], *just like for Ukrainians*, the EU flag is a symbol. These *two nations in the East are united* in their willingness to stand up for that flag and those values’ (15 March 2023). In that same debate, Vlad Gheorghe, a Romanian MEP from Renew Europe (RE), also uses strongly normative language to shame EU leaders: ‘The people of Georgia are fighting for *European values* and their weapons are the European flags. Their fight is non-violent, but they are very certain about their future. On the other hand, the Neighbourhood Commissioner is silent: he doesn’t fight for the Georgian people's right to be European’ (14 March 2023). The Romanian European People's Party (EPP) MEP Siegrid Muresan makes the equivalence even more explicit: ‘everything that we offer to Ukraine, we have to offer to the Republic of Moldova right now in terms of EU cooperation. In terms of perspective for membership, the Republic of Moldova and Ukraine are equally important for the security of Europe’ (12 November 2023).

This attempt to shame reluctant MEPs and EU leaders into supporting the enlargement prospects of their preferred candidates can also be found in instances beyond the Eastern Partnership candidates. For instance, a speech by Stéphane Séjourné, a French MEP (RE), highlights the need for consistency in the treatment of Ukraine and the Western Balkans: We cannot ‘ignore (…) the bloodshed in the name of the ideals of peace, democracy, and sovereignty in this unjust war… I hope that the same sense of responsibility will also guide the members of the Council on the issue of the Western Balkans’ (22 June 2022). The fact that the Western Balkans remain so prominent in enlargement discourse and that MEPs attempt to create equivalence in treatment with Kyiv provides in itself some support for entrapment, as it shows how the argument can be used to make the case for candidates that do not seem so similar to Ukraine. Overall, we detect some evidence of MEPs using the values of the EU to ‘trap’ decision-makers into extending Ukrainian support to other candidates.^9^ Whilst the effectiveness of this strategy when it comes to achieving actual accession for these countries remains to be seen (as discussed below), it is a strategy that has borne some fruits in the past (Schimmelfennig, 2001).

### Ideological orientations and support for enlargement

We expected MEPs’ ideological orientations to shape their discourse in the post-invasion period, with an increasing divide over positions towards enlargement between mainstream and radical parties. Specifically, we anticipated that radical party groups, and in particular the radical right, to respond with a strategy of ‘avoidance’ (De Vries and Hobolt, 2020) to the increasing public support for enlargement following Russia's invasion of Ukraine. Given the tension between their traditional scepticism towards enlargement and the shift in public opinion, we expected MEPs from these party groups to talk less about the issue without however necessarily changing their positions. In contrast, we expected MEPs from mainstream EPGs to speak both more and more positively about enlargement in the wake of Russia's aggression.

**Figure 3.**
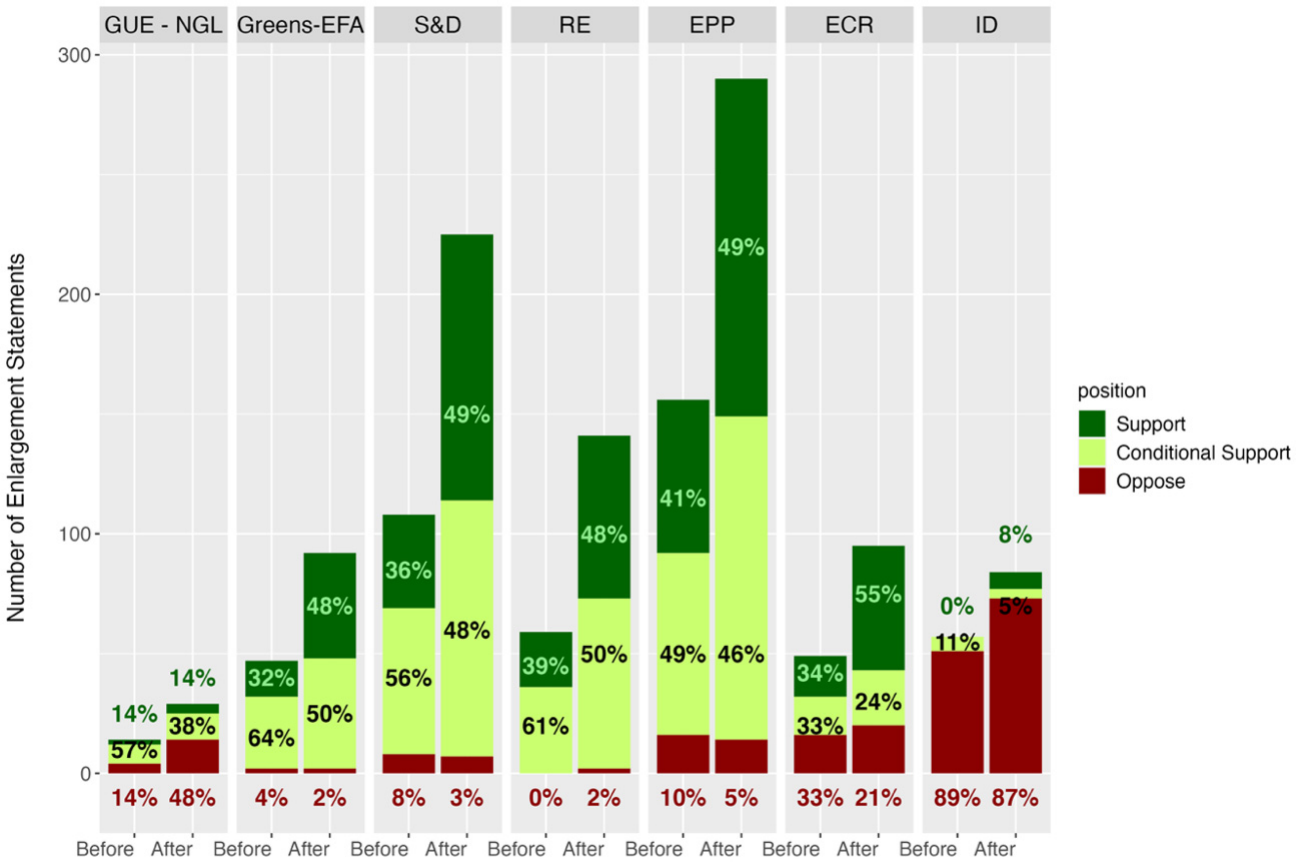
Position by party family before and after the Russian invasion.

*Note*: Percentage labels refer to share of enlargement statements in the respective investigation period (before and after the invasion) coded as ‘Oppose’, ‘Conditional Support’, and ‘Support’.

Figure 3 provides some evidence for this contrasting effect of ideology upon MEPs’ enlargement discourse. It plots the number and position of enlargement statements for each party family, showing that radical parties display the lowest increase in enlargement statements. This is particularly obvious for the radical right Identity and Democracy (ID), with an increase of 47% overall, compared to the number of enlargement statements more than doubling for RE and S&D. Their positions also do not seem to change drastically: ‘oppose’ is still the mode for ID (note that the Online appendix also provides the mean sentiment for each party family before and after the invasion), and for the radical left Confederal Group of the European United Left/Nordic Green Left (GUE/NGL), the mode actually changes from ‘conditional support’ to ‘oppose’, suggesting their discourse is in fact becoming even more sceptical. We note an interesting shift for the soft Eurosceptic European Conservatives and Reformists (ECR) group, for which the number of statements increases and the discourse becomes more positive, mirroring the pattern found for mainstream party groups. Unlike prior to the Russian invasion, where ECR was clearly sceptical towards further accessions (Wunsch and Bélanger, 2023), the ideological divide on enlargement in the EP now seems to run between the most extreme parties and the very large group of more centrist MEPs.

To test these hypotheses statistically, we again estimate several ordered logistic regression models, run the models separately for mainstream party groups, radical party groups (GUE-NGL and ID) and the radical right only (ID). We first run the models without candidate country random effects, and then introduce them. The results show that it is mainstream party groups that have changed their discourse in a significant way (hypothesis 2a). In contrast, the post-invasion coefficient for radical party groups is insignificant (hypothesis 2b). We conclude that whilst overall the need for consistency benefiting candidates beyond Ukraine is present, it is limited in its scope and excludes parties that have historically opposed enlargement to the EU. These findings lend support to our second set of hypotheses (2a to 2c) regarding the differences in how mainstream and radical party groups responded to Russia's invasion of Ukraine to adapt their enlargement discourse (Table 3).

**Table 3.** Party analysis – regression results with random effects (DV = Position).

	Mainstream parties	Radical parties	Radical right	Mainstream parties	Radical parties	Radical right
Post-invasion	0.44***	0.89	1.08	0.32*	0.94	1.08
	(0.16)	(0.63)	(0.92)	(0.18)	(0.69)	(0.93)
Observations	1262	184	141	1262	184	141
Speaker RE	Yes	Yes	Yes	Yes	Yes	Yes
Party FE	Yes	Yes	No	Yes	Yes	No
Candidate country RE	No	No	No	Yes	Yes	Yes

*p < 0.1; **p < 0.05; ***p < 0.01.

A qualitative analysis of our dataset focusing on the temporal variation of radical party groups’ enlargement statements illuminates how the strategy of avoidance unfolds. In the immediate months after the invasion, it is noticeable how radical right representatives still aim to shape the debate by urging caution over granting Ukraine candidate status. For instance, Belgian ID MEP Tom Vandendriessche sought to link Ukraine to other, less popular candidate countries: ‘As if that were not enough, after Albania and Turkey, they now also want to make Ukraine a member of the European Union. That country does not meet any of the necessary conditions. The Copenhagen criteria require a well-functioning market economy and a stable democratic rule of law. Ukraine has neither, but is the most corrupt country in Europe after Russia’ (15 June 2022).

As pro-Ukraine public opinion became consolidated however, we observe that these parties simply stop talking about Ukraine altogether. Indeed, after June 2022, ID MEPs have made only eight references to Ukraine (fewer than any other party), despite there being several plenary debates organised on the country. In the debate around the motion ‘One year of Russia's invasion and war of aggression against Ukraine’ on 15 February 2023, neither ID nor GUE/NGL made any statements whatsoever. In the debate on Ukraine's accession two weeks prior (2 February 2023), ID equally remained silent and we recorded a single short statement from GUE/NGL by Irish MEP Mick Wallace who criticised Zelensky's labour reforms. In contrast, mainstream pro-European parties took this opportunity to make an emotional case for enlargement based on the values of the community. For instance, Thijs Reuten, a Dutch MEP from the S&D group, stated that ‘The citizens of Kyiv, Pristina and Tirana are Europeans. They want their democracies protected, want clean air, fair wages, freedom of choice, fundamental rights. They want a better life. Let us live up to the promise of Europe – for ourselves, for all Europeans’ (28 February 2024).

When radical right party groups do occasionally engage on the issue of enlargement post-invasion, they do so to speak about other candidate countries, with their positions remaining equally hostile as prior to Russia's attack. Speaking about Kosovo's accession prospects, French ID MEP Jean-Lin Lacapelle described the country as ‘a pseudo-criminal and mafia state plagued by Islamism, which you [the EU] have made your darling child’ (9 May 2023). Hence, whilst radical party groups may have avoided the issue of enlargement post-invasion, there is little sign of them shifting their opposing positions. This observation is especially relevant in light of the radical right's electoral gains at the latest EP elections in June 2024.

One final interesting empirical detail is the extent to which new MEPs enter the enlargement debate post-invasion. Overall, we find that 240 MEPs spoke on enlargement after the invasion and 223 before. Of these, 101 made at least one statement on enlargement before and after the invasion. This shows that the share of repeat MEPs is relatively small and that the changes we see are associated in particular with new MEPs from certain parties entering the debate (and others opting out of it).

### Geographic origin and support for enlargement

**Figure 4.**
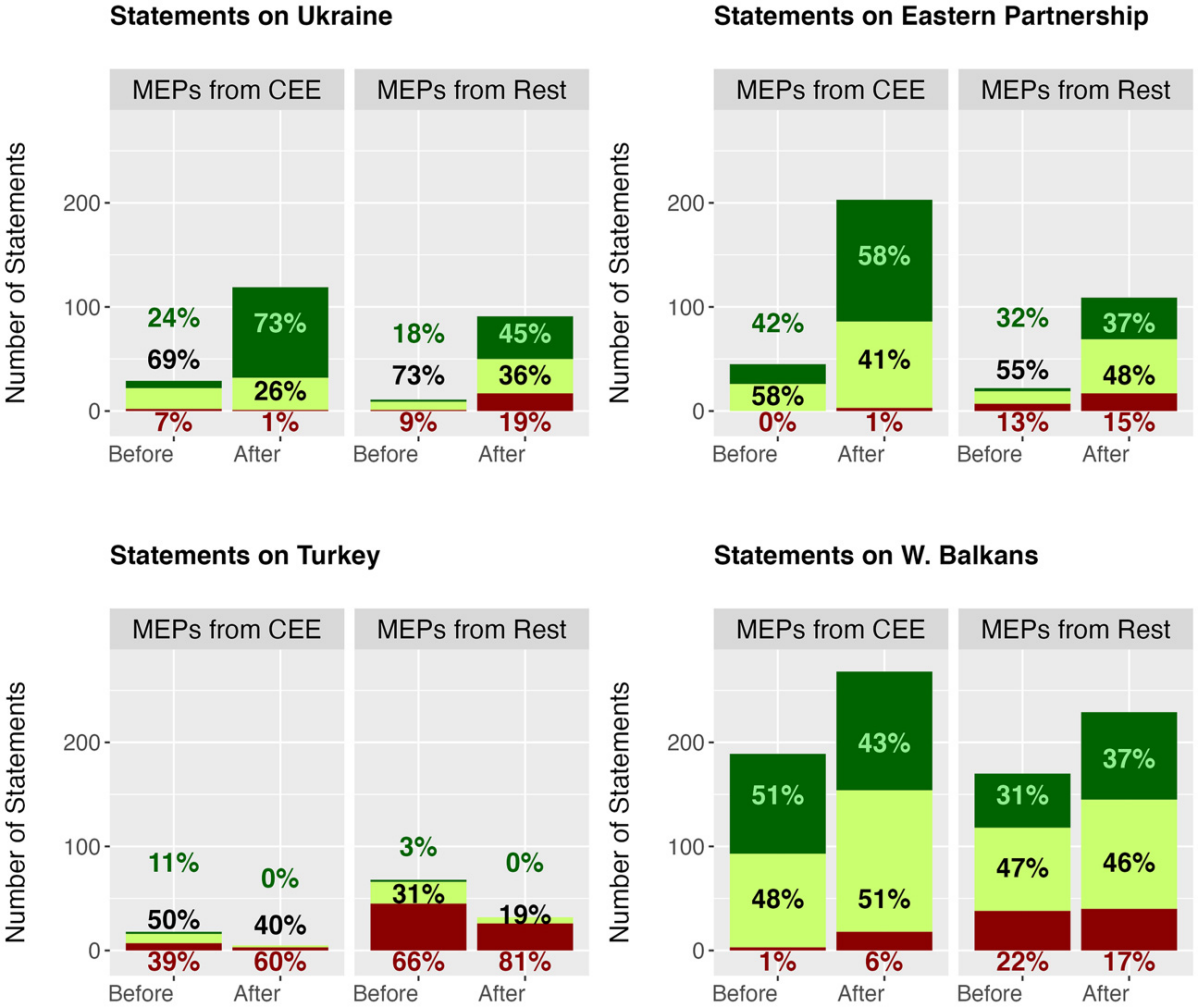
Position of MEPs by region before and after the Russian invasion.

*Note*: CEE refers to Central and Eastern Europe, rest refers to the rest of the EU, that is, MEPs from Northern, Southern, and Western Europe. Percentage labels refer to share of enlargement statements in the respective investigation period (before and after invasion) coded as ‘Oppose’ (red for bar and label), ‘Conditional Support’ (light green for bar, black for label), and ‘Support’ (dark green for bar and label). MEP: Members of the European Parliament; EU: European Union.

Finally, we consider differences is discourse based on MEPs’ country of origin, investigating whether the increasing support for a widening of the EU observed in the wake of Russia's full-scale invasion is driven by the traditionally more pro-enlargement Central and Eastern European MEPs. Figure 4 plots the enlargement speeches made before and after 24 February 2022, contrasting MEPs from the 11 CEE countries that entered the EU between 2004 and 2013 from the others. Whilst we focus here on distinguishing between the different (groups of) candidate countries under consideration, the Online appendix also provides an aggregate overview of all statements on enlargement, before and after the invasion (Figure A1). The recorded patterns clearly show how the increase in salience is driven by CEE MEPs. This group already dominated enlargement discourse before the invasion but this tendency is even more pronounced after Russia's aggression. Indeed, the number of statements recorded for CEE MEPs increased by 42% after the invasion, whereas for MEPs from the remaining countries the number of statements increased by 34%. In the Online appendix, we also include a breakdown of statements by country (Figure A2). These show interestingly how the more supportive positions post-invasion are shared across CEE countries, with the exception of the Czech Republic. It also shows how MEPs from France in particular remain negative, due to the predominance of French ID MEPs (Rassemblement National) involved in enlargement debates.

Besides the increase in salience, the increase in support in enlargement discourse is also most pronounced for CEE MEPs, although this depends on the candidate being discussed. Table 4 runs ordinal logistic regressions with an interaction term between an MEP's region of origin (CEE vs. rest) and our post-invasion independent variable. This shows how CEE MEPs, compared to MEPs from the rest of Europe, are significantly more supportive of Ukraine and Eastern Partnership candidates after the invasion. However, the interaction term is not statistically significant for the Western Balkans or Turkey. CEE MEPs also often highlight their historic ties with candidates when expressing their support. Joachim Stanislaw (ECR), for example, states that ‘Poland and Georgia are countries that have a centuries-old, very close cooperation – that is why Georgia is Poland's priority partner in the South Caucasus region’ (13 December 2022). The Polish MEP Andrzej Halicki (EPP) also links the prospects of existing candidates with the Eastern enlargement experience: ‘He [Putin] is afraid that Ukrainians will choose the same path that we, Poles, or our neighbours, Slovaks, Hungarians, or Romanians have chosen: democracy, civil rights, rapid development, and security, including security guaranteed by NATO’ (16 February 2022).

**Table 4.** Region of origin analysis: regression results with random effects (DV = position).

	Ukraine	Eastern Partnership	W. Balkans	Turkey
Post-invasion	0.04	0.58	1.44**	−0.27***
	(0.78)	(0.60)	(0.61)	(0.09)
CEE MEP	−1.74*	−0.82	1.04*	0.30**
	(0.93)	(0.73)	(0.61)	(0.14)
Post-invasion × CEE MEP	2.81***	1.74**	−0.65	0.04
	(0.98)	(0.73)	(0.76)	(0.22)
Observations	252	581	203	123
Party RE	Yes	Yes	Yes	Yes
Speaker RE	Yes	Yes	Yes	Yes

*Note*: The reference category for all models is MEPs from the rest of the EU (non-CEE).CEE: Central and Eastern Europe; MEP: Members of the European Parliament; EU: European Union.

*p < 0.1; **p < 0.05; ***p < 0.01.

More generally, a qualitative analysis of our statements reveals that MEPs from CEE countries attempt to use rhetorical action to link progress on enlargement to the EU's reputation and credibility. As stated by Vladimír Bilčík, an EPP MEP from Slovakia: ‘As we face Russia's attack against Ukraine, the EU must focus on its foundations. All of us inside the European Union, must once again become serious about enlargement… and equally deliver on our long-term commitment to the Western Balkans […] As Russia continues to pursue its hybrid warfare across the Western Balkans, we, as Europeans, must be politically clear. We will do what it takes to make Europe prevail in this region, to make democracy trump authoritarianism’ (22 November 2022). A similar sentiment is voiced by Andrius Kubilius, a Lithuanian MEP also from the EPP, in the same debate: ‘It's good that recently we have heard ambitious statements about enlargement towards Ukraine, Moldova and Western Balkans…Let us bring back the hope to our neighbours that the EU was able to wake up from its geopolitical laziness and I am absolutely sure that those neighbours will deliver major reforms of their countries. That is how enlargement can bring peace and prosperity for the whole European continent’ (22 November 2022). Overall, our analysis thus also provides support for our third set of hypotheses related to MEPs’ countries of origin, with the increase in salience (H3a) and support (H3b) for enlargement post-invasion most pronounced for CEE MEPs, albeit with the caveat that the increase in support primarily concerns Ukraine and Eastern Partnership candidates.

## Discussion and conclusion

Our study set out to explore the relevance of ‘rhetorical action’ in the context of renewed support for enlargement in the wake of Russia's full-scale aggression of Ukraine. Specifically, we examined whether increased political support for Ukraine's membership perspective has been used as an opportunity to push for a more generalised reinvigoration of the EU's enlargement policy by invoking normative arguments and the need for consistent treatment of the remaining candidate countries. Analysing plenary debates in the EP, we showed a clear increase in both the salience of enlargement and the support expressed by MEPs for the admission of further member states. This pattern extends beyond Ukraine to benefit in particular the other Eastern Partnership countries that share Kyiv's European ambitions, but also the Western Balkans. Geographic proximity and domestic support for the accession process thus appear as important scope conditions for the effectiveness of rhetorical action as a way to expand support for enlargement to larger groups of political actors. We show that these dynamics are primarily due to a renewed discursive commitment to the enlargement process among mainstream party groups and driven particularly by MEPs from Central and Eastern European member states. Rather than Ukraine ‘stealing the limelight’ from more long-standing candidate countries, Kyiv's current prominence and the political support it has received from the EU since February 2022 have at least temporarily contributed to a broader revitalisation of the EU's enlargement process.

Our findings shed light on the discursive dynamics inside the EP as a crucial institution for the accession process in a context of heightened geopolitical tensions between Moscow and Brussels. We are able to show that Russia's large-scale invasion of Ukraine corresponds to a rhetorical turning point in enlargement debates, inversing an earlier tendency of growing hostility towards further widening (Wunsch and Bélanger, 2023) with a marked rise in discursive support not only for Ukraine as the primary victim of Russian aggression, but for the Eastern Partnership and Western Balkan countries more broadly. Contrary to earlier tendencies for populist radical right parties to pose an increasingly united front against further expansion of EU membership (Bélanger and Wunsch, 2022), we show that these parties now become silent on the enlargement issue, largely leaving the rhetorical playing field to mainstream, pro-enlargement actors who strategically invoke shared values to push for a clearer commitment on the part of the EU to the admission of new member states.

In practical terms, it remains an open question to what extent renewed discursive support will translate into concrete results in the ongoing accession negotiations with the Eastern European candidate countries. Some initial concrete steps have already taken place, with the December 2023 European Council meeting deciding to open accession negotiations with Ukraine and Moldova and to grant candidate status to Georgia. Still, it is unclear whether and especially when the current pro-enlargement climate will lead to an actual new round of accessions in the near or even mid-range future. Although Ukrainian President Zelensky has sought to obtain concrete reassurances regarding a timeframe for his country's accession, there is a clear understanding among EU policy-makers that membership for Ukraine requires both a stable resolution of the military confrontation on the ground and a prior institutional reform to prepare the EU for the admission of new members. Growing wariness among European publics towards the ongoing military confrontation in Ukraine as well as the rise in right-wing, typically more Russia-friendly voices in the EP as well as several member states may come to represent important obstacles to swift progress towards the admission of any of the current accession hopefuls.

Furthermore, the role of the EP in enlargement policy is arguably less important than those of the European Council and the Commission. Whilst the Commission tends to be a big supporter of enlargement, it needs to weigh member states’ positions when developing its recommendations. The most recent enlargement decisions have been increasingly shaped by bilateral concerns individual member states bring to the process, considerably slowing the pace of membership negotiations (for instance Greece and Bulgaria's reservations regarding North Macedonia that were unrelated to the country's effective degree of compliance with accession conditions). Exploring how the invasion impacted the enlargement discourse of these two other crucial EU institutions would be valuable avenue for future research.

On the whole, the return of war on Europe's soil and the meanwhile blatantly obvious geopolitical nature of EU enlargement have created a window of opportunity for a more decisive engagement in supporting the political, economic, and social transformations required to ready the EU's Eastern neighbours for eventual membership. At the same time, the very complexity of these processes, in addition to the strong emphasis on internal reform as a precondition for further widening, mean that the path is still long for Ukraine as well as its neighbours. Whilst rhetorical entrapment was key in forcing member states to follow their words with deeds back in 2004, the political climate has since become more volatile, making a repeat ‘big bang’ enlargement under the impression of an overarching historical narrative less likely.

## Supplemental Material

sj-docx-1-eup-10.1177_14651165251367355 - Supplemental material for A geopolitical turning point? Enlargement discourse after the Russian invasion of Ukraine Supplemental material, sj-docx-1-eup-10.1177_14651165251367355 for A geopolitical turning point? Enlargement discourse after the Russian invasion of Ukraine by Tom Hunter, Natasha Wunsch and Marie-Eve Bélanger in European Union Politics

sj-zip-2-eup-10.1177_14651165251367355 - Supplemental material for A geopolitical turning point? Enlargement discourse after the Russian invasion of Ukraine Supplemental material, sj-zip-2-eup-10.1177_14651165251367355 for A geopolitical turning point? Enlargement discourse after the Russian invasion of Ukraine by Tom Hunter, Natasha Wunsch and Marie-Eve Bélanger in European Union Politics

sj-zip-3-eup-10.1177_14651165251367355 - Supplemental material for A geopolitical turning point? Enlargement discourse after the Russian invasion of Ukraine Supplemental material, sj-zip-3-eup-10.1177_14651165251367355 for A geopolitical turning point? Enlargement discourse after the Russian invasion of Ukraine by Tom Hunter, Natasha Wunsch and Marie-Eve Bélanger in European Union Politics

## Notes

1. EU enlargement has occasionally proven divisive also within the main party families in the EP – see Wunsch and Bélanger (2023).

2. These include the European People's Party (EPP), the Socialists and Democrats (S&D), Renew Europe (RE), and the Greens.

3. We include here Identity and Democracy (ID), the European Conservatives and Reformists (ECR), and the Left Group (GUE/NGL).

4. There are some nuances worth mentioning. For instance, Hungary tends to be more supportive of the Western Balkans whereas Poland and the Baltic states tend to be more supportive of Ukraine and other Eastern Partnership countries (see e.g. Buras and Morina, 2023).

5. The nesting of clusters is hierarchical for the models in Tables 2 and 4 as speakers are grouped within parties. The models in Table 3 assume a crossed random effects structure, as there is no strict hierarchical relationship between speakers and candidate countries.

6. To be clear, this article will treat Ukraine and the other Eastern Partnership countries separately, so any mentions of the Eastern Partnership in figures (e.g. Figures 1 and 3) or in tables (e.g. Table 2) do not include Ukraine.

7. The Online appendix includes the regression tables from this section with additional slope estimates and threshold coefficients (see Tables A2 and A3).

8. It is worth noting here that grouping together candidates from the Western Balkans and to a lesser extent the Eastern Partnership candidates (in Figure 1 and Table 2) does mask some cross-country differences. We do so to provide a digestible overview but acknowledge this limitation and that further research could explore these differences between candidates within regional enlargement waves.

9. Interestingly, our dataset has 40 instances of MEPs mentioning both Ukraine and a Western Balkans candidate. Out of these, 31 are positive statements supporting Ukraine, and of these 31 supportive statements, 30 also support the Western Balkan candidate. This again showcases the difficulty of supporting Ukraine without extending that support to other candidates.

## References

[bibr1-14651165251367355] Anghel V Džankić J (2023) Wartime EU: Consequences of the Russia–Ukraine war on the enlargement process. Journal of European Integration 45(3): 487–501.

[bibr2-14651165251367355] Anghel V Jones E (2023) Is Europe really forged through crisis? Pandemic EU and the Russia–Ukraine war. Journal of European Public Policy 30(4): 766–786.

[bibr3-14651165251367355] Azrout R Van Spanje JH Vreese D , et al. (2013) Focusing on differences? Contextual conditions and anti-immigrant attitudes’ effects on support for Turkey’s EU membership. International Journal of Public Opinion Research 25(4): 480–501.

[bibr4-14651165251367355] Bélanger M Schimmelfennig F (2021) Politicization and rebordering in EU enlargement: Membership discourses in European parliaments. Journal of European Public Policy 28(3): 407–426.

[bibr5-14651165251367355] Bélanger M Wunsch N (2022) From cohesion to contagion? Populist radical right contestation of EU enlargement. Journal of Common Market Studies 60(3): 653–672.

[bibr6-14651165251367355] Braghiroli S (2023) Europe’s Russia-friendly parties put to the test by Putin’s invasion of Ukraine. Journal of Regional Security 18(1): 29–38.

[bibr7-14651165251367355] Brel-Fournier Y Morrison MK (2021) The predicament of Europe’s ‘last dictator’. International Area Studies Review 24(3): 169–192.

[bibr8-14651165251367355] Buras P Morina E (2023) Catch-27: The contradictory thinking about enlargement in the EU. European Council on Foreign Relations Policy Brief. Available at: https://ecfr.eu/publication/catch-27-the-contradictory-thinking-about-enlargement-in-the-eu/ (accessed 14 July 2025).

[bibr9-14651165251367355] De Vries C Hobolt S (2020) Political Entrepreneurs: The Rise of Challenger Parties in Europe. Princeton, NJ: Princeton University Press.

[bibr10-14651165251367355] Dixon JC (2010) Opposition to enlargement as a symbolic defence of group position: Multilevel analyses of attitudes toward candidates’ entries in the EU-25. The British Journal of Sociology 61(1): 127–154. 10.1111/j.1468-4446.2009.01305.x 20377600

[bibr11-14651165251367355] Economides S (2020) *From fatigue to resistance: EU enlargement and the Western Balkans*. Working Paper (17). Berlin: The Dahrendorf Forum.

[bibr12-14651165251367355] Economides S Featherstone K Hunter T (2024) The changing discourses of EU enlargement: A longitudinal analysis of national parliamentary debates. Journal of Common Market Studies 62(1): 168–185.

[bibr13-14651165251367355] Economist . (2023) The EU is finally rebooting the enlargement machine. Available at: https://www.economist.com/europe/2023/09/28/the-eu-is-finally-rebooting-the-enlargement-machine (accessed 14 July 2025)

[bibr14-14651165251367355] Elster J (1992) Local Justice: How Institutions Allocate Scarce Goods and Necessary Burdens. New York: Russell Sage Foundation.

[bibr15-14651165251367355] European Council of Foreign Relations . (2023) New poll: Europeans open to Ukraine joining the EU despite security risks, but cool on further enlargement of the bloc ahead of crucial European Council summit. Available at: https://ecfr.eu/publication/europeans-open-to-ukraine-joining-the-eu-despite-security-risks/ (accessed 14 July 2025)

[bibr16-14651165251367355] Feliu L Serra F (2015) The European Union as a ‘normative power’ and the normative voice of the European Parliament. In: The European Parliament and its International Relations. London: Routledge, 17–34.

[bibr17-14651165251367355] Financial Times . (2023) Balkans’ frustration mounts over Ukraine’s fast-track to EU membership. Available at: https://www.ft.com/content/b654fe14-25b0-44f3-bea6-96a8596d6cde (accessed 14 July 2025)

[bibr18-14651165251367355] Genschel P Leek L Weyns J (2023) War and integration. The Russian attack on Ukraine and the institutional development of the EU. Journal of European Integration 45(3): 343–360.

[bibr19-14651165251367355] Green-Pedersen C Mortensen PB (2015) Avoidance and engagement: Issue competition in multiparty systems. Political Studies 63(4): 747–764.

[bibr20-14651165251367355] Håkansson C (2024) The Ukraine war and the emergence of the European Commission as a geopolitical actor. Journal of European Integration 46(1): 25–45.

[bibr21-14651165251367355] Heinisch R Werner A Habersack F (2020) Reclaiming national sovereignty: The case of the conservatives and the far right in Austria. European Politics and Society 21(2): 163–181.

[bibr22-14651165251367355] Hix S Noury A Roland G (2005) Power to the parties: Cohesion and competition in the European Parliament, 1979–2001. British Journal of Political Science 35(2): 209–234.

[bibr23-14651165251367355] Hobolt SB Tilley J (2018) Fleeing the centre: The rise of challenger parties in the aftermath of the euro crisis. In: Europe’s Union in Crisis. London: Routledge, 57–77.

[bibr24-14651165251367355] Hoffmann I (2023) Anxious we stand: despite worries, Europeans remain steadfast in support for Ukraine. Bertelsmann Stiftung Europe. Available at: https://globaleurope.eu/europes-future/anxious-we-stand-despite-worries-europeans-remain-steadfast-in-support-for-ukraine/ (accessed 14 July 2025).

[bibr25-14651165251367355] Hooghe L Marks G (2005) Calculation, community and cues. European Union Politics 6(4): 419–443.

[bibr26-14651165251367355] Hunter T (2025) Disintegration and party competition: Evidence from parliamentary speeches on Brexit. Journal of European Public Policy 32(7): 1573–1596.

[bibr27-14651165251367355] Ivaldi G Zankina E (2023) *ECPS report: the impacts of the Russian invasion of Ukraine on right-wing populism in Europe*. European Center for Populism Studies. Available at: https://www.populismstudies.org/ecps-report-the-impact-of-the-russia-ukraine-war-on-right-wing-populism-in-europe/ (accessed 14 July 2025).

[bibr28-14651165251367355] Kriesi H (2016) The politicization of European integration. Journal of Common Market Studies 54: 32–47.

[bibr29-14651165251367355] Leuffen D Dimitrova AL Sedelmeier U , et al. (2024) Rhetorical action in a liberal international order in crisis: Theorising EU and NATO enlargements post-2022. Journal of European Public Policy: 1–46. DOI: 10.1080/13501763.2024.2413461.

[bibr30-14651165251367355] Martini M Walter S (2024) Learning from precedent: How the British Brexit experience shapes nationalist rhetoric outside the UK. Journal of European Public Policy 31(5): 1231–1258. 38868721 10.1080/13501763.2023.2176530 PMC11166053

[bibr31-14651165251367355] McLaren LM (2007) Explaining opposition to Turkish membership of the EU. European Union Politics 8(2): 251–278.

[bibr32-14651165251367355] Newnham R (2020) Russia and Belarus: Economic linkage in a patron–client relationship. Journal of Belarusian Studies 9(1): 3–26.

[bibr33-14651165251367355] O’Brennan J (2014) ‘On the slow train to nowhere?’ The European Union, ‘enlargement fatigue’ and the Western Balkans. European Foreign Affairs Review 19(2): 221–241.

[bibr34-14651165251367355] Panchuk D (2024) The impact of the Russian invasion of Ukraine on public support for EU enlargement. Journal of European Public Policy 31(10): 3128–3150.

[bibr35-14651165251367355] Scherzinger J (2023) Unbowed, unbent, unbroken? Examining the validity of the responsibility to protect. Cooperation and Conflict 58(1): 81–101.

[bibr36-14651165251367355] Schimmelfennig F (2001) The community trap: Liberal norms, rhetorical action, and the Eastern enlargement of the European Union. International Organization 55(1): 47–80.

[bibr37-14651165251367355] Schimmelfennig F (2021) Rhetorical entrapment in EU–Turkey relations. In: EU–Turkey Relations: Theories, Institutions, and Policies. Cham: Springer, 139–156.

[bibr38-14651165251367355] Stolle D (2024) Aiding Ukraine in the Russian war: Unity or new dividing line among Europeans? European Political Science 23: 218–233.

[bibr39-14651165251367355] Szolucha A (2010) The EU and ‘enlargement fatigue’: Why has the European Union not been able to counter ‘enlargement fatigue’? Journal of Contemporary European Research 6(1): 107–122.

[bibr40-14651165251367355] Toshkov D Kortenska E Dimitrova A Fagan A , et al. (2014) The ‘old’ and the ‘new’ Europeans: Analyses of public opinion on EU enlargement in review. In: Has the EU’s Eastern Enlargement Brought Europe Together?. Netherlands: MAXCAP Working Paper Series, 1–42.

[bibr41-14651165251367355] Von der Leyen (2023) Keynote speech by President von der Leyen at the Ukraine Recovery Conference 2023. Available at: https://enlargement.ec.europa.eu/news/keynote-speech-president-von-der-leyen-ukraine-recovery-conference-2023-2023-06-21_en (accessed 6 August 2025).

[bibr42-14651165251367355] Wunsch N Bélanger M (2023) Radicalisation and discursive accommodation: Responses to rising Euroscepticism in the European Parliament. West European Politics 47(6): 1223–1250.

[bibr43-14651165251367355] Wunsch N Olszewska N (2022) From projection to introspection: Enlargement discourses since the ‘big bang’ accession. Journal of European Integration 44(7): 919–939.

[bibr44-14651165251367355] Yougov . (2022) Les Européens sont-ils favorables à l'adhésion de l'Ukraine à l'UE? Available at: https://fr.yougov.com/politics/articles/41747-les-europeens-favorables-adhesion-de-lukraine (accessed 14 July 2025)

[bibr45-14651165251367355] Zuur A Ieno E Walker N , et al. (2009) Mixed effects modelling for nested data. In: Mixed Effects Models and Extensions in Ecology with R. New York: Springer, 101–142.

