# Supplementary material for: A geopolitical turning point? Enlargement discourse after the Russian invasion of Ukraine
Source: Eur Union Polit. 2025 Aug 21;26(4):689–713. doi: 10.1177/14651165251367355 (PMC12619659; doi:10.1177/14651165251367355)
Supplement: sj-docx-1-eup-10.1177_14651165251367355 - Supplemental material for A geopolitical turning point? Enlargement discourse after the Russian invasion of Ukraine [file sj-docx-1-eup-10.1177_14651165251367355.docx]

**Online appendix**

**A geopolitical turning point? Enlargement discourse after the Russian invasion of Ukraine**

**Authors:**

Tom Hunter, Natasha Wunsch and Marie-Eve Bélanger

**Table of contents**

Section A1: Figures

- Figure A1: CEE vs. Rest of EU member states comparison
- Figure A2: Position of MEPs by member state: EP Ninth Session before and after Russian invasion (24 Feb 2022)

Section A2: Regression Models

- Table A1: ICC, AIC and BIC for random effects and fixed effects models
- Table A2: Ordered Logistic Regression Results with slope estimates
- Table A3: Party Analysis with slope estimates and threshold coefficients
- Table A4: Mean Sentiment by party family before and after the invasion

**Section A1: Figures**


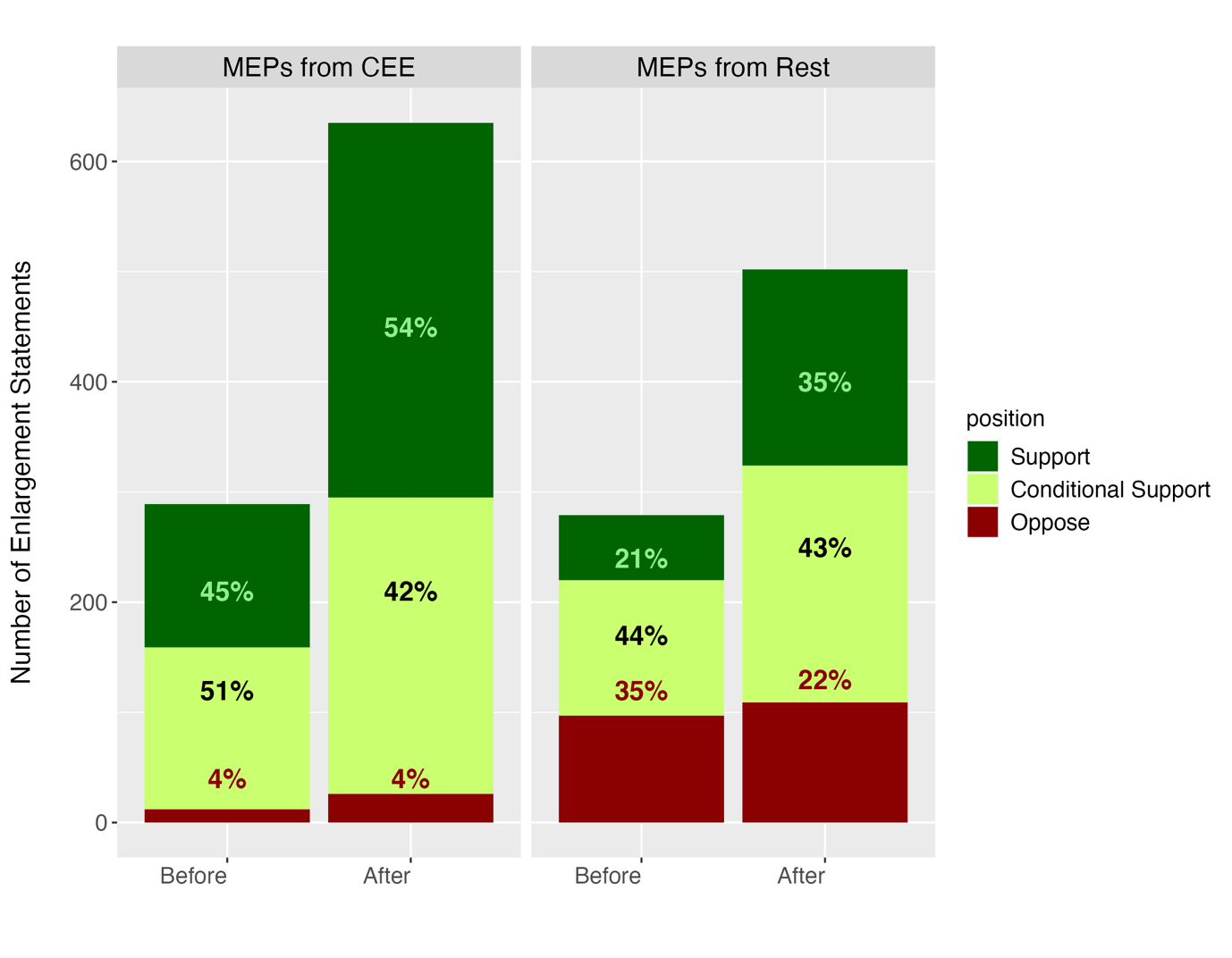


Figure A1 - CEE vs. Rest of EU member states comparison (Aggregated sample)

*Note: CEE refers to Central and Eastern Europe, Rest refers to the rest of the EU, i.e. MEPs from Northern, Southern and Western Europe. Percentage labels refer to share of enlargement statements in the respective investigation period (Before and After the Invasion) coded as ‘Oppose’ (red for bar and label), ‘Conditional Support’ (light green for bar, black for label), and ‘Support’ (dark green for bar and label).*


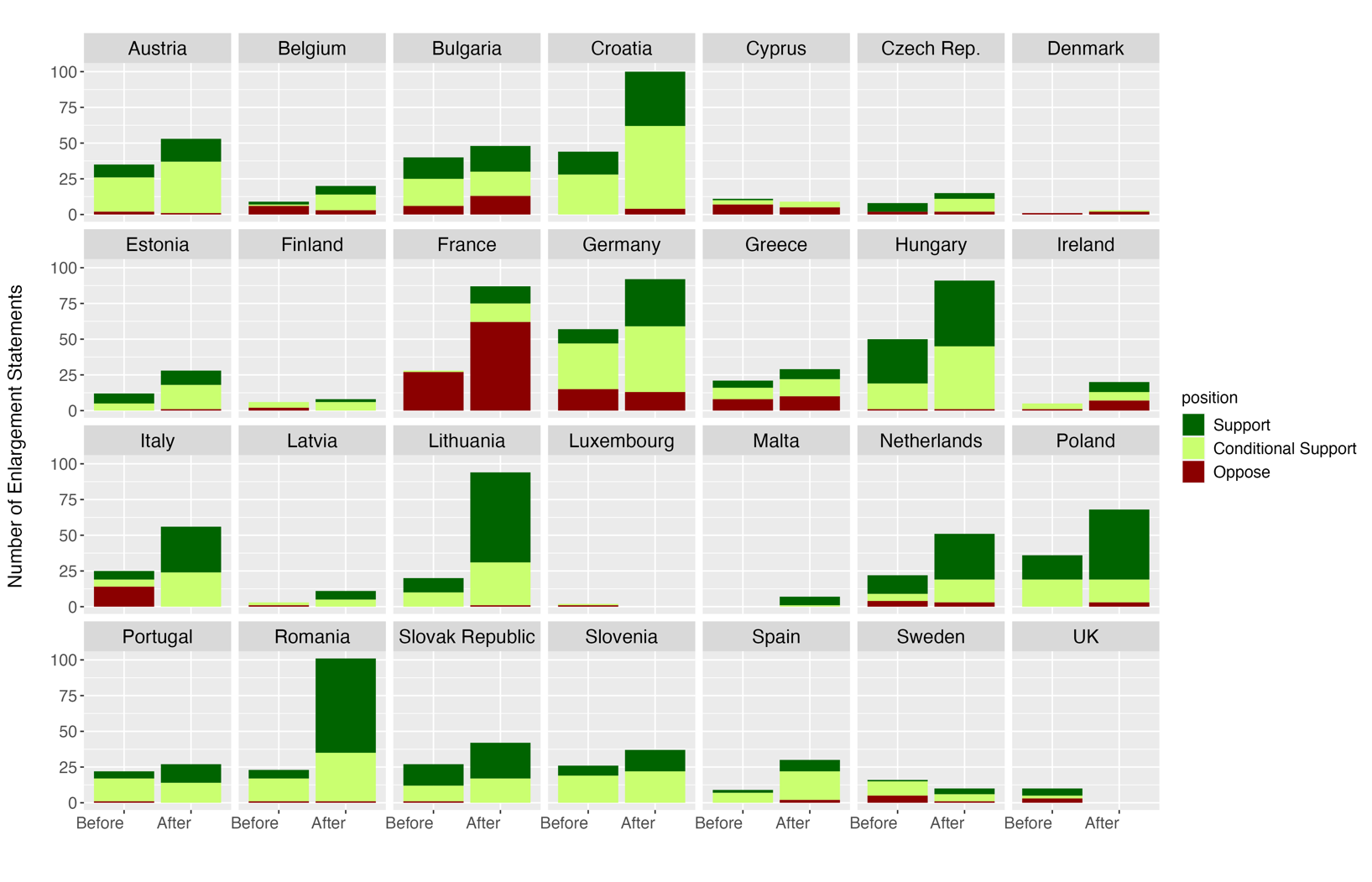


Figure A2. Position of MEPs by member state: EP Ninth Session before and after Russian invasion (24 Feb 2022)

**Section A2: Regression Models**

To test the appropriateness of random effect models vis-a-vis a simpler fixed effects models, we run for each of the models in Tables 2, 4, and 5 a simpler model that excludes the random effects and compare both the AIC and BIC scores for the fixed effect only model and the model with random effects. As Table A1 below shows, in each case the random effects model has significantly lower AIC and BIC scores, which means that the inclusion of random effects is indeed appropriate and improves the model fit.

Table A1: ICC, AIC and BIC for random effects and fixed effects models

| **Model** | **Adjusted ICC** | **AIC - With random effects** | **AIC - Fixed effects only** | **BIC - With random effects** | **BIC - Fixed effects only** |
| --- | --- | --- | --- | --- | --- |
| Table 2, Model 1 | 0.696 | 2515.003 | 3183.299 | 2541.892 | 3199.432 |
| Table 2, Model 2 | 0.717 | 2218.661 | 2895.406 | 2310.083 | 2976.073 |
| Table 2, Model 3 | 0.671 | 2320.568 | 2951.966 | 2379.723 | 3000.365 |
| Table 4, Mainstream Parties (First model) | 0.536 | 2062.287 | 2251.880 | 2103.411 | 2287.863 |
| Table 4, Radical Parties (First model) | 0.769 | 186.2011 | 225.6428 | 202.2758 | 238.5026 |
| Table 4, Radical Right (First model) | 0.943 | 96.0506 | 132.4458 | 107.8456 | 141.2921 |
| Table 4, Mainstream Parties (Second model) | 0.619 | 1816.24 | 2251.880 | 1862.504 | 2287.863 |
| Table 4, Radical Parties (Second model) | 0.806 | 186.7135 | 225.6428 | 206.0031 | 238.5026 |
| Table 4, Radical Right (Second model) | 0.943 | 98.04315 | 132.4458 | 112.7870 | 141.2921 |
| Table 5. Ukraine | 0.626 | 364.1889 | 425.6693 | 388.8949 | 443.3164 |
| Table 5. EP | 0.631 | 835.2842 | 986.9187 | 865.8375 | 1008.742 |
| Table 5. W Balkans | 0.471 | 319.3315 | 340.8946 | 342.5240 | 357.4606 |
| Table 5. Turkey | 0.732 | 182.1038 | 184.1275 | 201.7891 | 198.1884 |

Table A2: Ordered Logistic Regression Results with slope estimates and threshold coefficients


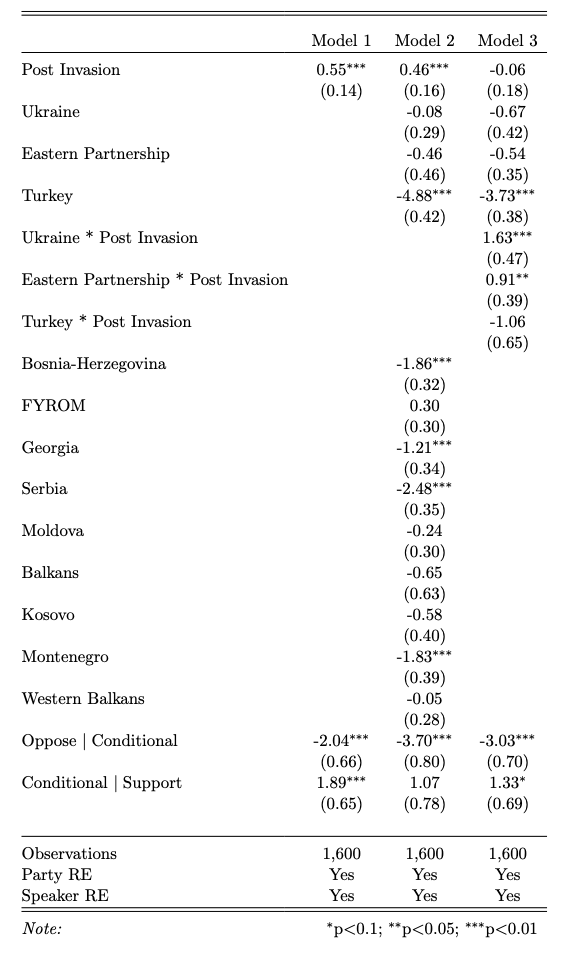


Table A3: Party Analysis with slope estimates and threshold coefficients


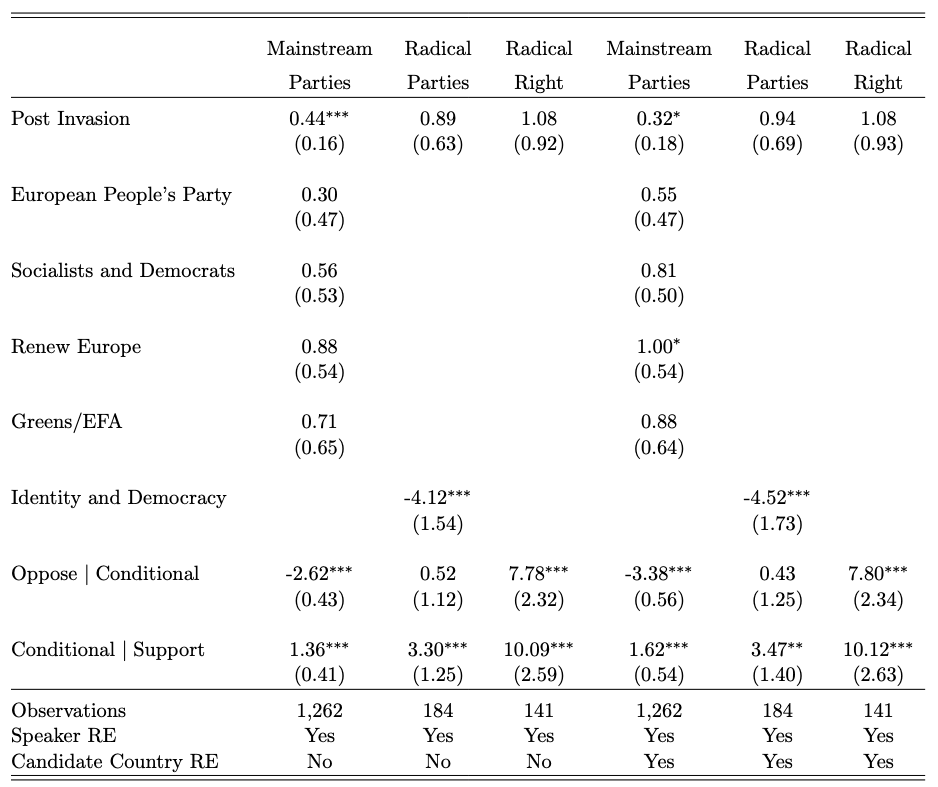


Note: ECR is the reference category for Mainstream Parties. GUE – NG is the reference category for Radical Parties. *p<0.1; **p<0.05; ***p<0.01

Table A4: Mean Sentiment by party family before and after the invasion

| **Party Family** | **Mean Sentiment Before** | **Mean Sentiment After** |
| --- | --- | --- |
| GUE – NGL | 1.86 | 1.66 |
| Greens – EFA | 2.28 | 2.46 |
| S&D | 2.29 | 2.46 |
| RE | 2.39 | 2.47 |
| EPP | 2.31 | 2.44 |
| ECR | 2.02 | 2.34 |
| ID | 1.11 | 1.21 |

Note: Mean sentiment calculated with following values: Oppose = 1; Conditional Support = 2; Support = 3
